# Strategies for Referring Cancer Patients in a Smoking Cessation Program

**DOI:** 10.3390/ijerph17176089

**Published:** 2020-08-21

**Authors:** James M. Davis, Leah C. Thomas, Jillian E. H. Dirkes, H. Scott Swartzwelder

**Affiliations:** 1 Duke Department of Medicine, Duke Cancer Institute, Duke University, Durham, NC 27707, USA; 2 Duke Cancer Institute, Duke University, Durham, NC 27707, USA; leah.thomas@duke.edu (L.C.T.); jillian.dirkes@duke.edu (J.E.H.D.); 3 Duke Department of Psychiatry and Behavioral Sciences, Duke University, Durham, NC 27707, USA; hss@duke.edu

**Keywords:** smoking cessation, electronic health record, program utilization, referral methods

## Abstract

Most people who smoke and develop cancer are unable to quit smoking. To address this, many cancer centers have now opened smoking cessation programs specifically designed to help cancer patients to quit. An important question has now emerged—what is the most effective approach for engaging smokers within a cancer center in these smoking cessation programs? We report outcomes from a retrospective observational study comparing three referral methods—traditional referral, best practice advisory (BPA), and direct outreach—on utilization of the Duke Cancer Center Smoking Cessation Program. We found that program utilization rate was higher for direct outreach (5.4%) than traditional referral (0.8%), *p* < 0.001, and BPA (0.2%); *p* < 0.001. Program utilization was 6.4% for all methods combined. Inferring a causal relationship between referral method and program utilization was not possible because the study did not use a randomized design. Innovation is needed to generate higher utilization rates for cancer center smoking cessation programs.

## 1. Introduction

### 1.1. Smoking Cessation in People with Cancer

Treatment of tobacco dependence is uniquely important in people with cancer. In the United States, smoking causes approximately 28.6% of all cancer deaths [1] and causes multiple types of cancer, including lung, laryngeal, pharyngeal, esophageal, pancreatic, bladder, stomach, colorectal, liver, cervical, renal, and acute myeloid leukemia [2]. Quitting smoking has been shown to decrease the chances of developing the most common forms of lung cancer—small cell, large cell, squamous cell, and adenocarcinoma [3]. Smoking cessation after a cancer diagnosis is associated with increased survival in lung cancer [4] and head and neck cancers [5]. Smoking cessation also leads to a reduced risk of cancer recurrence in lung cancer [6], breast cancer [7], bladder cancer [8], and gastric cancer [9]. Smoking cessation decreases side effects related to cancer treatment, leads to a better quality of life, and leads to a lower risk of developing another primary cancer [7]. Smoking is associated with poor wound healing, an increased rate of surgical wound infections [10], and post-surgical pulmonary complications [11].

A diagnosis of cancer is widely considered to be a teachable moment—a somewhat rare opportunity that arises in a person’s life when major behavioral change is possible [12]. That said, the National Health and Nutrition Examination Survey (NHANES) study showed that only 36.1% of smokers quit smoking after a cancer diagnosis [13]. Another study showed that 44% of smokers quit smoking after their cancer diagnosis and that only 62% received smoking cessation counseling from their medical providers [14]. A survey of the American Society of Clinical Oncology found that 84% of oncologists advise their patients to quit smoking, but only 39% provide smoking cessation support [15]. Barriers to smoking cessation reported by people with cancer include stress related to their cancer diagnosis, a desire to maintain personal control, and limited understanding o how smoking impacts cancer treatment and outcomes [16]. The National Cancer Institute (NCI) has now mandated that every NCI-designated cancer center provide an onsite smoking cessation program to help people with cancer to quit smoking [17,18].

### 1.2. Specialized Smoking Cessation Treatment for People with Cancer 

There is a now a wide variety of evidence-based smoking cessation treatments available, including seven FDA-approved smoking cessation medications [19], and an array of evidence-based behavioral treatments [20,21]. Guidelines describing evidence-based smoking cessation treatment are routinely disseminated throughout health systems [22,23], and the National Comprehensive Cancer Network Tobacco Treatment Guideline provides evidence-based treatment approaches for smokers with cancer [24]. NCI also leads an initiative to support NCI-designated cancer centers in developing smoking cessation services [25]. Provider training for specialized smoking cessation programs is now available through tobacco treatment specialist training programs provided at over 20 sites across the US [26,27]. Established cancer center smoking cessation programs have demonstrated biochemical smoking abstinence rates of over 30% [28,29], roughly ten times as high as abstinence rates in an unassisted quit attempt [30].

### 1.3. Patient Engagement in Smoking Cessation Services 

**Figure 1:**
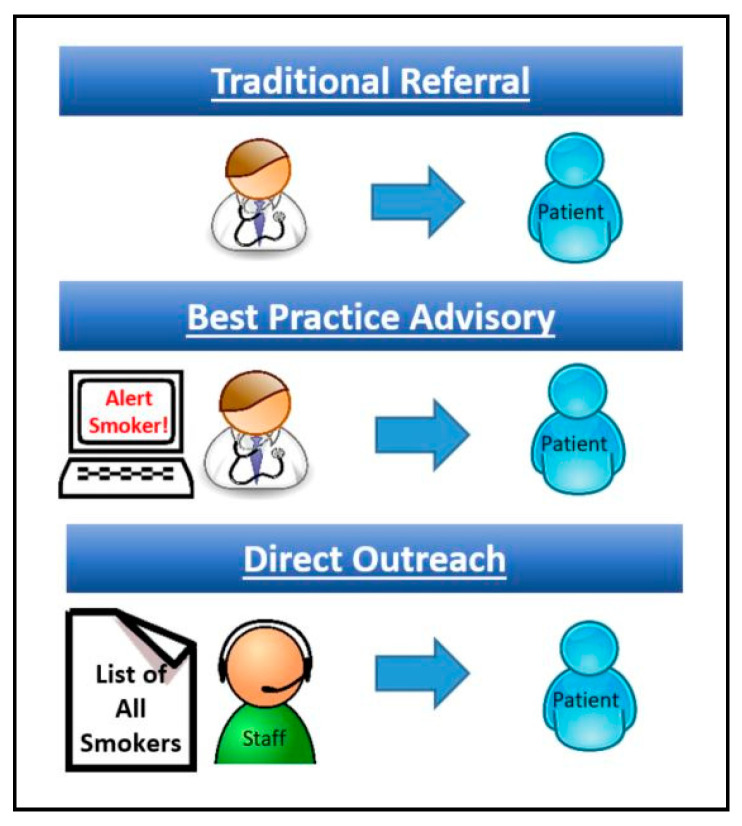
Referral methods.

A key step in helping cancer patients to quit smoking is referral to a smoking cessation program. We discuss three methods of patient referral in this paper—traditional referral, best practice advisory (BPA), and direct outreach (Figure 1). The default method of referral in any institution is an unassisted provider referral. This occurs, for example, when a medical provider decides that their patient might benefit from seeing another provider and then refers their patient. This widely practiced, unassisted form of referral, which we call here traditional referral, has the advantage of arising from a patient–provider relationship and helps to maintains a degree of treatment continuity [31]. Unfortunately, traditional referral is limited by a provider’s busy schedule and the need to manage other medical problems [32]. Another approach is use of the best practice advisory (BPA). BPA is like traditional referral, but it includes an alert from the electronic health record letting the provider know that their patient would benefit from a particular treatment [33]. BPA outcomes have often been quite modest, because providers who are busy with other issues routinely ignore these BPA alerts [34]. A specific form of BPA—the opt-out referral—appears to be more promising. In a BPA with an opt-out referral, the BPA fires and the referral order is placed automatically unless the provider cancels it [35]. A small study on breast cancer patients using opt-out referral showed an increase in program utilization from 12% (9/75) to 56% (15/27) [36]. A third approach, direct outreach, provides proactive population-based outreach by phone, email, text or mail to patients with a particular problem and then offer services for that problem [37]. One study on automated direct outreach to cancer center smokers showed increased utilization of a smoking cessation program from 5.8% to 20.2% [38]. The purpose of this study is to use observational data to compare three referral methods on utilization of a cancer center smoking cessation program.

## 2. Materials and Methods

We conducted a retrospective observational study on data from patients referred to the Duke Cancer Center Smoking Cessation Program by traditional referral and BPA over a 1-year period between 1 October 2017 and 30 September 2018. In addition, we conducted pilot testing of direct outreach from 1 July to 31 July 2018. We then used these data to compare all three referral methods—traditional referral, best practice advisory, and direct outreach—on referral rates and program utilization. Primary outcomes for this study were program referral rate (the portion of smokers attending visits at Duke Cancer Center who were referred to the Duke Cancer Center Smoking Cessation Program) and program utilization rate (the portion of cancer center smokers who attended an appointment at the Duke Cancer Center Smoking Cessation Program). Data were analyzed using descriptive analyses and chi-square for comparison of groups. This study was supported by funding from the National Cancer Institute, grant supplement P30-CA014236-43S3. The study was approved by the Duke University Health System Institutional Review Board. As a retrospective observational study on aggregate patient outcomes of routine clinical care, the study did not require notification of or consent from patients or providers.

### 2.1. Program Referral

The Duke Cancer Center Smoking Cessation Program employs a team-based approach with collaboration of medical and behavioral providers trained through the Duke-UNC Tobacco Treatment Specialist Training Program. The Duke Cancer Center Smoking Cessation Program exclusively serves the Duke Cancer Center. Current tobacco users who receive care of any kind (cancer screening, chemotherapy, radiation, surgery, etc.) at Duke Cancer Center may be referred to the Duke Cancer Center Smoking Cessation Program from any Duke Cancer Center provider (oncologist, radiation oncologist, surgical oncologist, etc.). Cancer center providers can refer their patients to the Duke Smoking Cessation Program by placing an order in the electronic health record. In addition, cancer center providers received a BPA (alert in the electronic health record) on all of their patients who were current smokers, informing them that their patient was a current smoker, and presenting an electronic link to refer their patient to the program. Cancer center providers were not required to make a program referral and only made a referral if this arose naturally during cancer care. Cancer center providers were unaware that an observational study was being conducted to assess referral outcomes. As such, providers referred patients as they normally do during routine clinical care. Direct outreach was not conducted by medical providers and was conducted by program staff.

### 2.2. Program Utilization

Once a patient is referred, they are contacted by a Program Coordinator who describes the program and assists in triage and scheduling. Referral outcomes collected include the following: *no answer*, *patient declined*, *call back later*, and *appointment scheduled*. All referrals receive a minimum of three call attempts. Patients who decline services are offered referral to QuitlineNC, a national telephonic tobacco quitline [39]. Quitline outcomes were not assessed for this report. After an appointment is scheduled, patients sometimes *cancel* or *reschedule*; and patients will sometimes be a *no show* to an appointment. Patients receive a phone call from a Program Coordinator six months after their first scheduled appointment regardless of whether they attended. If that patient is continuing to use tobacco, program services are offered again. A small number of patients received more than one form of referral; these were patients who agreed to an appointment through direct outreach, but their providers also placed a traditional referral. This occurred in 2 patients—one was counted as a traditional referral, the other as direct outreach.

### 2.3. Traditional Referral

**Figure 2:**
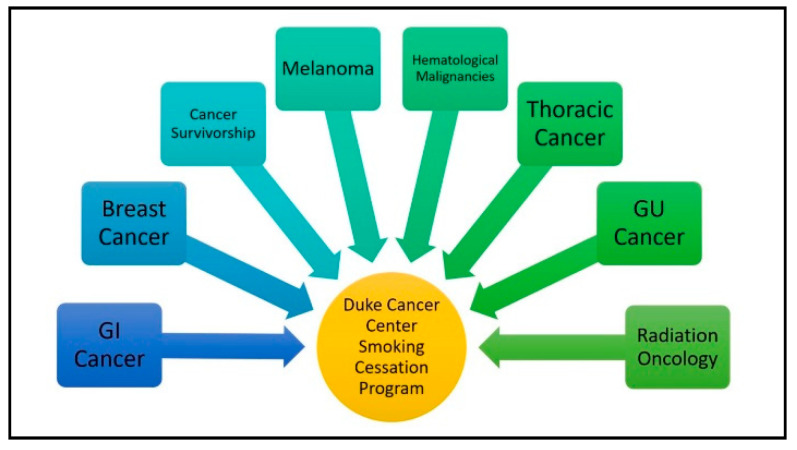
Traditional referral to the Duke Cancer Center Smoking Cessation Program.

Figure 2 shows traditional referral occurring from multiple cancer center departments to the Duke Cancer Center Smoking Cessation Program. To maximize the use of traditional referrals, smoking cessation providers presented information on the smoking cessation program at oncology provider meetings, staff meetings, and staff events. Of the 8 groups listed in Figure 2, we conducted informational meetings with physicians and separate meetings with staff for each group. All clinics (providers and staff) received information on traditional referral and BPA prior to the data collection period and then received this information again at some point during the data collection period. A total of 17 presentations about the smoking cessation program were provided at the Duke Cancer Center prior to the 12-month data collection period.

### 2.4. Best Practice Advisory

In our program, BPA is opt-in, not opt-out, such that the provider must open the BPA alert and place a referral. The alert does not provide a hard stop and may be ignored in order to manage other issues. Nursing staff are encouraged to click the BPA and are able to create a pended order, which can then be signed by the medical provider. To maximize the use of BPA, we provided instructions at all provider and staff presentations. In most cases, training on how to open the BPA alert occurred at the same time as training on how to place a program referral. The BPA build was not time-consuming for our technical staff, and BPAs are commonly used at Duke for other purposes.

### 2.5. Direct Outreach

We conducted a 30-day pilot study on direct outreach. For this study, we generated a list from the electronic health record showing all tobacco users with an appointment at the Duke Cancer Center in the next 30 days and then called these patients by phone to offer services. If there was no response, patients were sent an electronic message through the electronic health record patient communication portal. Direct outreach was conducted by a social worker and a medical assistant, both with training as tobacco treatment specialists. These staff members are intimately familiar with the program and capable of addressing patient questions with a degree of sophistication. The two providers spent 60 h (3 h/day × 20 days) making outreach calls.

### 2.6. Data Collection and Analysis

#### 2.6.1. Population-Based Tobacco Use

Duke requires that every patient’s smoking history is updated by nursing staff at each clinical visit. Patients in our program were identified as smokers by accessing the smoking history component of the electronic health record. Duke requires that smoking history be updated for each patient by nursing staff at each clinical visit, although there are currently no data on how well nursing staff complies with this requirement.

#### 2.6.2. Referral Database

Data on program referrals and appointment attendance were collected through a referral database in EPIC, an electronic health record system. BPA: Data on BPA outcomes were obtained from an electronic health record dataset designed to track BPA referrals. Direct Outreach: Direct outreach was piloted over a 30-day period with program referral and utilization outcomes tracked on each patient through the pilot study database. Clinical Outcomes: Data entry within the Duke Cancer Center Smoking Cessation Program is algorithmic, such that key data elements are entered into the electronic health record at every visit. Data collected include smoking history, cancer diagnosis, psychiatric history, previous smoking cessation treatment, and carbon monoxide breath testing to assess smoking abstinence and reduction. Analysis: Data were analyzed using descriptive analyses and chi-square for comparison of groups. Data on traditional referral and BPA were compared using 12-month datasets. Data on direct outreach were compared to other referral methods using 30-day datasets. Data presented here are based on unique patients; repeat visits for individuals are not included so as to avoid double counting.

## 3. Results

### 3.1. Demographics

In the 12-month assessment period, the roughly 150 Duke Cancer Center clinicians (e.g., medical, surgical, and radiation oncologists) cared for over 50,000 patients, including 6040 smokers (approximately a 12% rate of current smoking). Of these, patients who attended an appointment with the Duke Cancer Center Smoking Cessation Program had a mean age of 53.8 (SD 10.1); 43.7% were female, 28.1% were African American, 65.6% were Caucasian, and 6.3% were other/unknown/not reported race. Primary insurance providers were 56.3% Medicare/Medicaid, 31.2% private insurance, and 12.5% self-pay.

### 3.2. 12-Month Program Referral and Utilization

Over the 12-month period, 6040 current smokers received care of any kind (cancer screening, chemotherapy, radiation, surgery, etc.) by any Duke Cancer Center provider (oncologist, radiation oncologist, surgical oncologist, etc.). Duke Cancer Center providers had access through the electronic health record to place an order for their patients to be referred to the Duke Cancer Center Smoking Cessation Program. In addition, cancer center providers received a BPA (alert in the electronic health record) on all of their patients who were current smokers, informing them that their patient was a current smoker, and presenting an electronic link to refer patients to the program. The referral rate over a 12-month period was defined as the total number of patients who were referred to the Duke Cancer Center Smoking Cessation Program divided by 6040—the total number of current smokers seen by any provider at the Duke Cancer Center. During the 12-month assessment period, the Duke Cancer Center Smoking Cessation Program received a total of 230 referrals, a referral rate of 3.8% (230/6040). Traditional referral led to 183 of these referrals (3% referral rate) and was significantly higher than BPA, which led to 47 referrals (0.8% referral rate); X*^2^* (1, *N* = 6039) = 61.4, *p* < 0.001. The program utilization rate over a 12-month period was defined as the total number of patients who attended an appointment at the Duke Cancer Center Smoking Cessation Program divided by the total number of current smokers seen at Duke Cancer Center. During the 12-month assessment period, 99 unique patients attended the Duke Cancer Center Smoking Cessation Program for a program utilization rate of 1.6% (99/6040). Of these 99 program attendees, traditional referral resulted in 91 attendees (1.5% utilization rate), significantly higher than BPA, which resulted in 8 program attendees (0.1% utilization rate); X*^2^* (1, *N* = 6039) = 68.5, *p* < 0.001. The chance of interaction between referral methods (e.g., BPA reducing traditional referrals) was low due to low overall utilization rates (e.g., 0.1% × 1.5% = 0.15% possible interaction).

### 3.3. 30-Day Pilot Test—Program Referral and Utilization

**Figure 3:**
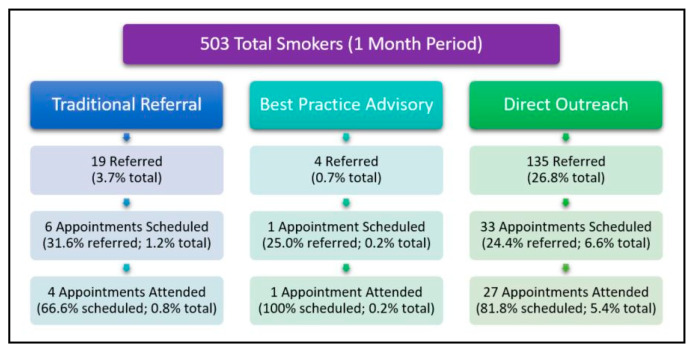
Referral methods (1 month).

Over the 30-day period (1 July–30 July 2018), we pilot-tested direct outreach to assess program referral rate and utilization rate for this referral method (Figure 3). During this 30-day period, 503 current smokers received care of any kind (cancer screening, chemotherapy, radiation, surgery, etc.) by all Duke Cancer Center providers (oncologist, radiation oncologists, surgical oncologist etc.). The referral rate over this 30-day period was defined as the total number of patients who were referred to the Duke Cancer Center Smoking Cessation Program divided by 503—the total number of current smokers seen for any reason at the Duke Cancer Center. Over this 30-day period, there were a total of 158 referrals to the Duke Cancer Center Smoking Cessation Program, a referral rate of 31.4% (158/503). Of the 158 patients who were referred, direct outreach led to referral of 135 (26.8% referral rate), traditional referral led to referral of 19 (3.7% referral rate), and BPA led to referral of 4 patients (0.7% referral rate). Although direct outreach appears to show a higher referral rate than other methods, we did not conduct a statistical comparison, because these referral methods are managed differently (direct outreach referrals include scheduling of patient appointments during the referral phone call, whereas traditional referral and BPA lead to scheduling of patient appointments during a future phone call). The program utilization rate over this 30-day pilot testing period was defined as the total number of patients who attended an appointment at the Duke Cancer Center Smoking Cessation Program divided by 503—the total number of current smokers seen for any reason at the Duke Cancer Center. Over this 30-day period, a total of 32 patients attended a visit at the Duke Cancer Center Smoking Cessation Program, a program utilization rate of 6.4% (32/503). Of these 32 program attendees, direct outreach led to 27 (5.4% utilization rate), traditional referral led to 4 (0.8% utilization rate), and BPA led to 1 attendee (0.2% utilization rate). During the 30-day pilot test, program utilization rate for direct outreach was significantly higher than for traditional referral; X*^2^* (1, *N* = 502) = 16.1, *p* < 0.001. Differences in program utilization between traditional referral and direct outreach were not significant, but the numbers were so small (1 attendee vs. 4 attendees) that statistical comparison was not meaningful.

### 3.4. Smoking Outcomes

Carbon monoxide (CO) breath testing was collected at all clinic visits to assess smoking abstinence [40], but self-reported smoking rates were not available. Reduction in smoking was assessed via change in expired breath CO alone, with baseline CO of 17.8 (SD = 15.4), and final visit CO of 11.3 (SD = 12.2). This represented a 36.5% reduction of CO over the treatment (mean 17.82). The timing of clinic visits to the smoking cessation program was not uniform across the sample but coordinated with cancer center treatment visits.

## 4. Discussion

### 4.1. Main Findings

A comparison of the three referral methods showed that direct outreach led to higher rates of program referral and program utilization than traditional referral, and that traditional referral was associated with higher rates of program utilization than BPA. Although differences in program utilization between these referral methods were fairly large, it was not possible to infer a causal relationship between referral method and program utilization because patients were not randomized to referral methods. An important conclusion from these outcomes is that traditional referral alone may be insufficient for recruitment of patients into a cancer center smoking cessation program. This is an important finding because most smoking cessation programs at least begin by relying on traditional referral as the primary means of referral. Our findings suggest that, if possible, efforts should be made to expand methods of patient outreach beyond traditional referral.

### 4.2. Low Referral and Utilization Rates

A commonly expressed critique of smoking cessation programs is that all outcomes appear to be low. Although there is a need for improvement, it is important to recognize that at any given time, the percentage of smokers who are willing to make a quit attempt is typically only 14–20% [41,42,43,44]. An important consideration is that many smokers who are unwilling to make a quit attempt at one point in time may be willing to make a quit attempt if asked again at a later date [45,46]. This suggests that there is likely merit in reaching out to smokers on multiple occasions over time. Another important consideration is the growing understanding that smokers who are unwilling to make a quit attempt often benefit from engaging in tobacco use treatment initially focused on smoking reduction, and that this approach can lead to smoking cessation [47].

### 4.3. Traditional Referral within a Cancer Center

Traditional referral led to a program utilization rate of only 1.5% (12-month data) of all smokers receiving services at the Duke Cancer Center. One reason for this low referral rate could be that oncology providers tend to be busy and often do not feel that there is sufficient time to discuss smoking cessation while providing cancer treatment services [48]. This low program utilization rate is also attributable in part to attrition. Over 12 months, 183 smokers were referred for smoking cessation services through traditional referral, but only 91 (35.8%) attended an appointment. This is likely indicative of the large number of barriers that the cancer population faces, including financial, transportation, scheduling, fatigue, and illness due to cancer or cancer treatment [16]. Overcoming these multiple barriers will require multiple strategies. The Duke Cancer Center Smoking Cessation program is currently attempting to address some of these barriers by providing patients with access to remote smoking cessation treatment at home through text-based programs [49], app-based programs [50], web-based programs [51], and phone-based programs [39].

### 4.4. Best Practice Advisory (BPA)

Although, this study was not randomized and provided no assurance regarding bias, an unexpected finding was that BPA had quite a small effect (0.2% for 12 months) on program utilization. The most common explanation for the relatively low use of the BPA is alert fatigue, in which an electronic health record presents so many alerts that providers ignore them in order to complete more pressing work [34,52]. Studies have now described an inverse relationship between the number of alerts presented in an electronic health record and the chance that the provider will open an alert [34]. Navigation of this challenging situation is not simple. Education of physicians within our institution showed temporary increases in BPA use, but use of BPAs remained low. Nursing staff may be more likely to respond to BPAs than physicians [53]. To capture this opportunity, BPAs may be targeted to nurses who may place a pended referral order to be countersigned by the physician. Alternately, a BPA may be configured as opt-out, such that the order goes through for a physician signature unless specifically declined [54].

### 4.5. Direct Outreach

Although this study provided no assurance regarding bias, direct outreach appeared to be associated with higher program utilization. The major challenge to implementation of direct outreach methods within a smoking cessation program is that manual phone calls (our approach) were time-consuming. One solution to this is to develop automated forms of direct outreach. Automated outreach can be conducted through interactive voice response [55], email and text messaging [56], and messaging through the electronic health record [57]. The Duke Cancer Center Smoking Cessation Program is exploring several of these methods to enhance utilization of our services.

### 4.6. Limitations

This study had several limitations. This was a retrospective observational study and patients were not randomized to various referral methods. In a non-randomized trial, it would be impossible to exclude various forms of bias. Another limitation is that the study used two databases (referral and clinical) that did not allow for integration of patient-level data; as such, it was not possible to report smoking abstinence for each referral method. This limitation could be overcome in a prospective analysis. The study also included a wide range of patients with varying degrees of illness and barriers to treatment (mental illness, financial challenges, and/or stages of cancer) such that results may not generalize to healthier portions of the general population. Additionally, we provided data on direct outreach for only a 30-day period, with less stability than 12-month outcomes.

## 5. Conclusions

Smoking cessation is uniquely important for smokers who have cancer; it improves cancer treatment outcomes, reduces cancer recurrence, and reduces overall mortality. It has now been recognized as necessary that cancer centers provide specialized smoking cessation treatment for smokers with cancer. We assessed three approaches to patient recruitment into the Duke Cancer Center Smoking Cessation Program and found that direct outreach (generating a list of all cancer patients who smoke and then calling these patients on the phone) led to the highest rates of program utilization. Traditional referral alone did not lead to high rates of program utilization, and best practice advisory showed rather poor outcomes. Innovative solutions are needed to increase utilization of cancer center smoking cessation programs.

## References

[1] Lortet-Tieulent J., Sauer A.G., Siegel R.L., Miller K.D., Islami F., Fedewa S.A., Jacobs E.J., Jemal A. (2016). State-Level Cancer Mortality Attributable to Cigarette Smoking in the United States. JAMA Intern. Med..

[2] Centers for Disease Control and Prevention (2020). Smoking Cessation: A Report of the Surgeon General.

[3] Khuder S.A., Mutgi A.B. (2001). Effect of smoking cessation on major histologic types of lung cancer. Chest.

[4] Ferketich A.K., Niland J.C., Mamet R., Zornosa C., D’Amico T.A., Ettinger D.S., Kalemkerian G.P., Pisters K.M., Reid M.E., Otterson G.A. (2013). Smoking status and survival in the national comprehensive cancer network non–small cell lung cancer cohort. Cancer.

[5] Gillison M.L., Zhang Q., Jordan R., Xiao W., Westra W.H., Trotti A., Spencer S., Harris J., Chung C.H., Ang K.K. (2012). Tobacco Smoking and Increased Risk of Death and Progression for Patients With p16-Positive and p16-Negative Oropharyngeal Cancer. J. Clin. Oncol..

[6] Tezel Y.B., Akyil M., Tezel C., Akyil F.T., Evman S., Gurer D., Baysungur V., Yalcinkaya I. (2016). Impact of persistence of smoking on recurrence after early stage lung surgery. Eur. Respir. J..

[7] Toll B.A., Brandon T.H., Gritz E.R., Warren G.W., Herbst R.S. (2013). AACR Subcommittee on Tobacco and Cancer Assessing tobacco use by cancer patients and facilitating cessation: An American Association for Cancer Research policy statement. Clin. Cancer Res..

[8] van Osch Frits H.M., Jochems Sylvia H.J., van Schooten Frederik J., Bryan Richard T., Zeegers Maurice P. (2016). Significant Role of Lifetime Cigarette Smoking in Worsening Bladder Cancer and Upper Tract Urothelial Carcinoma Prognosis: A Meta-Analysis. J. Urol..

[9] Praud D., Rota M., Pelucchi C., Bertuccio P., Rosso T., Galeone C., Zhang Z.-F., Matsuo K., Ito H., Hu J. (2018). Cigarette smoking and gastric cancer in the Stomach Cancer Pooling (StoP) Project. Eur. J. Cancer Prev..

[10] Sørensen L.T. (2012). Wound healing and infection in surgery. The clinical impact of smoking and smoking cessation: A systematic review and meta-analysis. Arch. Surg..

[11] Mills E., Eyawo O., Lockhart I., Kelly S., Wu P., Ebbert J.O. (2011). Smoking cessation reduces postoperative complications: A systematic review and meta-analysis. Am. J. Med..

[12] Dresler C., Warren G.W., Arenberg D., Yang P., Steliga M.A., Cummings K.M., Stone E., Jassem J. (2018). “Teachable Moment” Interventions in Lung Cancer: Why Action Matters. J. Thorac. Oncol..

[13] Tseng T.S., Lin H.Y., Moody-Thomas S., Martin M., Chen T. (2012). Who tended to continue smoking after cancer diagnosis: The national health and nutrition examination survey 1999–2008. BMC Public Health.

[14] Burke L., Miller L.-A., Saad A., Abraham J. (2009). Smoking Behaviors Among Cancer Survivors: An Observational Clinical Study. J. Oncol Pract..

[15] Warren G.W., Marshall J.R., Cummings K.M., Toll B.A., Gritz E.R., Hutson A., Dibaj S., Herbst R., Mulshine J.L., Hanna N. (2013). Addressing tobacco use in patients with cancer: A survey of American Society of Clinical Oncology members. J. Oncol. Pract..

[16] Wells M., Aitchison P., Harris F., Ozakinci G., Radley A., Bauld L., Entwistle V., Munro A., Haw S., Culbard B. (2017). Barriers and facilitators to smoking cessation in a cancer context: A qualitative study of patient, family and professional views. BMC Cancer.

[17] Cinciripini P.M., Karam-Hage M., Kypriotakis G., Robinson J.D., Rabius V., Beneventi D., Minnix J.A., Blalock J.A. (2019). Association of a comprehensive smoking cessation program with smoking abstinence among patients with cancer. JAMA Netw. Open.

[18] Morgan G., Schnoll R.A., Alfano C.M., Evans S.E., Goldstein A., Ostroff J., Park E.R., Sarna L., Cox L.S. (2011). National Cancer Institute conference on treating tobacco dependence at cancer centers. J. Oncol. Pract..

[19] Cahill K., Stevens S., Perera R., Lancaster T. (2013). Pharmacological interventions for smoking cessation: An overview and network meta-analysis. Cochrane Database Syst. Rev..

[20] Lancaster T., Stead L. Does Individually-Delivered Counselling Help People to Stop Smoking?. https://www.cochrane.org/CD001292/TOBACCO_does-individually-delivered-counselling-help-people-stop-smoking.

[21] Stead C.A., Lancaster T. Do Group-based Smoking Cessation Programmes Help People to Stop Smoking?. https://www.cochrane.org/CD001007/TOBACCO_do-group-based-smoking-cessation-programmes-help-people-stop-smoking.

[22] Center for Disease Control and Prevention (2014). Best Practices for Comprehensive Tobacco Control Programs—2014.

[23] Curry S.J., Keller P.A., Orleans C.T., Fiore M.C. (2008). The role of health care systems in increased tobacco cessation. Annu. Rev. Public Health.

[24] Shields P.G., Herbst R.S., Arenberg D., Benowitz N.L., Bierut L., Luckart J.B., Cinciripini P., Collins B., David S., Davis J. (2016). Smoking cessation, version 1.2016, NCCN clinical practice guidelines in oncology. J. Natl. Compr. Cancer Netw..

[25] D’Angelo H., Rolland B., Adsit R., Baker T.B., Rosenblum M., Pauk D., Morgan G.D., Fiore M.C. (2019). Tobacco Treatment Program Implementation at NCI Cancer Centers: Progress of the NCI Cancer Moonshot-Funded Cancer Center Cessation Initiative. Cancer Prev. Res..

[26] Council for Tobacco Treatment Training Programs. https://ctttp.org/accredited-programs/.

[27] Slattery C., Freund M., Gillham K., Knight J., Wolfenden L., Bisquera A., Wiggers J. (2016). Increasing smoking cessation care across a network of hospitals: An implementation study. Implement Sci..

[28] Karam-Hage M., Oughli H.A., Rabius V., Beneventi D., Wippold R.C., Blalock J.A., Cinciripini P.M. (2016). Tobacco cessation treatment pathways for cancer patients: 10 Years in the making models for smoking cessation practice. J. Natl. Compr. Cancer Netw..

[29] Hays J.T., Croghan I.T., Schroeder D.R., Burke M.V., Ebbert J.O., McFadden D.D., Hurt R.D. (2011). Residential treatment compared with outpatient treatment for tobacco use and dependence. Mayo Clin. Proc..

[30] Hughes J.R., Keely J., Naud S. (2004). Shape of the relapse curve and long-term abstinence among untreated smokers. Addiction.

[31] Nett L.M. (1990). The physician’s role in smoking cessation. A present and future agenda. Chest.

[32] Bains M., Britton J., Marsh J., Jayes L., Murray R.L. (2014). Patients’ and healthcare professionals’ views on a specialist smoking cessation service delivered in a United Kingdom hospital: A qualitative study. Tob. Induc. Dis..

[33] Lurio J., Morrison F.P., Pichardo M., Berg R., Buck M.D., Wu W., Kitson K., Mostashari F., Calman N. (2010). Using electronic health record alerts to provide public health situational awareness to clinicians. J. Am. Med. Inf. Assoc..

[34] Ancker J.S., Edwards A., Nosal S., Hauser D., Mauer E., Kaushal R. (2017). Effects of workload, work complexity, and repeated alerts on alert fatigue in a clinical decision support system. BMC Med. Inf. Decis. Mak..

[35] Faseru B., Ellerbeck E.F., Catley D., Gajewski B.J., Scheuermann T.S., Shireman T.I., Mussulman L.M., Nazir N., Bush T., Richter K.P. (2017). Changing the default for tobacco-cessation treatment in an inpatient setting: Study protocol of a randomized controlled trial. Trials.

[36] Nolan M., Ridgeway J.L., Ghosh K., Martin D., Warner D.O. (2019). Design, implementation, and evaluation of an intervention to improve referral to smoking cessation services in breast cancer patients. Support Care Cancer.

[37] Haas J.S., Linder J.A., Park E.R., Gonzalez I., Rigotti N.A., Klinger E.V., Kontos E.Z., Zaslavsky A.M., Brawarsky P., Marinacci L.X. (2015). Proactive tobacco cessation outreach to smokers of low socioeconomic status: A randomized clinical trial. JAMA Intern. Med..

[38] Giuliani M.E., Liu G., Xu W., Dirlea M., Selby P., Papadakos J., Abdelmutti N., Yang D., Eng L., Goldstein D.P. (2019). Implementation of a Novel Electronic Patient-Directed Smoking Cessation Platform for Cancer Patients: Interrupted Time Series Analysis. J. Med. Internet Res..

[39] NC Department of Health and Human Services QuitlineNC.com. https://www.quitlinenc.com/.

[40] Cropsey K.L., Trent L.R., Clark C.B., Stevens E.N., Lahti A.C., Hendricks P.S. (2014). How low should you go? Determining the optimal cutoff for exhaled carbon monoxide to confirm smoking abstinence when using cotinine as reference. Nicot. Tob. Res..

[41] Prochaska J.O. (1996). A stage paradigm for integrating clinical and public health approaches to smoking cessation. Addict. Behav..

[42] Campbell S., Bohanna I., Swinbourne A., Cadet-James Y., McKeown D., McDermott R. (2013). Stages of change, smoking behaviour and readiness to quit in a large sample of indigenous Australians living in eight remote north Queensland communities. Int. J. Environ. Res. Public Health.

[43] Glantz S., Kroon L., Prochaska J., Schroeder S. Smoking Cessation: Theory and Practice of Behavior Change (Lecture). https://medschool.ucla.edu/workfiles/Site-System/Teaching-Materials/53-Smoking-Cessation-LEC-2007.pdf.

[44] Etter J.-F., Sutton S. (2002). Assessing “stage of change” in current and former smokers. Addiction.

[45] Chaiton M., Diemert L., Cohen J.E., Bondy S.J., Selby P., Philipneri A., Schwartz R. (2016). Estimating the number of quit attempts it takes to quit smoking successfully in a longitudinal cohort of smokers. BMJ Open.

[46] Patel K.K., Jones P.G., Ellerbeck E.F., Buchanan D.M., Chan P.S., Pacheco C.M., Moneta G., Spertus J.A., Smolderen K.G. (2018). Underutilization of evidence-based smoking cessation support strategies despite high smoking dddiction burden in peripheral artery disease specialty care: Insights from the International PORTRAIT registry. J. Am. Heart Assoc..

[47] Robinson J., McEwen A., Heah R., Papadakis S. (2019). A ‘Cut-Down-To-Stop’ intervention for smokers who find it hard to quit: A qualitative evaluation. BMC Public Health.

[48] Keto J., Jokelainen J., Timonen M., Linden K., Ylisaukko-oja T. (2015). Physicians discuss the risks of smoking with their patients, but seldom offer practical cessation support. Subst. Abus. Treat. Prev. Policy.

[49] National Institute of Health Frequently Asked Questions–Smokefree Text. https://smokefree.gov/tools-tips/text-programs/faqs.

[50] Vilardaga R., Rizo J., Zeng E., Kientz J.A., Ries R., Otis C., Hernandez K. (2018). User-centered design of learn to quit, a smoking cessation smartphone app for people with serious mental illness. JMIR Serious Games.

[51] Davis J.M., Manley A.R., Goldberg S.B., Stankevitz K.A., Smith S.S. (2015). Mindfulness training for smokers via web-based video instruction with phone support: A prospective observational study. BMC Complement. Altern. Med..

[52] Kane-Gill S.L., O’Connor M.F., Rothschild J.M., Selby N.M., McLean B., Bonafide C.P., Cvach M.M., Hu X., Konkani A., Pelter M.M. (2017). Technologic Distractions (Part 1): Summary of approaches to manage alert quantity with intent to reduce alert fatigue and suggestions for alert fatigue metrics. Crit. Care Med..

[53] Drahnak D.M. (2018). An observational study of the effects of a clinical nurse specialist quality improvement project for clinical reminders for sepsis on patient outcomes and nurse actions. J. Emerg. Crit. Care Med..

[54] Schechter-Perkins E.M., Miller N.S., Hall J., Hartman J.J., Dorfman D.H., Andry C., Linas B.P. (2018). Implementation and Preliminary Results of an Emergency Department Nontargeted, Opt-out Hepatitis C Virus Screening Program. Acad. Emerg. Med..

[55] Rigotti N.A., Chang Y., Rosenfeld L.C., Japuntich S.J., Park E.R., Tindle H.A., Levy D.E., Reid Z.Z., Streck J., Gomperts T. (2017). Interactive voice response calls to promote smoking cessation after hospital discharge: Pooled analysis of two randomized clinical trials. J. Gen. Intern. Med..

[56] Scott-Sheldon L.A.J., Lantini R., Jennings E.G., Thind H., Rosen R.K., Salmoirago-Blotcher E., Bock B.C. (2016). Text Messaging-Based Interventions for Smoking Cessation: A Systematic Review and Meta-Analysis. JMIR Mhealth Uhealth.

[57] Ralston J.D., Rutter C.M., Carrell D., Hecht J., Rubanowice D., Simon G.E. (2009). Patient Use of Secure Electronic Messaging Within a Shared Medical Record: A Cross-sectional Study. J. Gen. Intern. Med..

